# Concordance between decision analysis and matching systematic review of randomized controlled trials in assessment of treatment comparisons: a systematic review

**DOI:** 10.1186/1472-6947-14-57

**Published:** 2014-07-15

**Authors:** Rahul S Mhaskar, Hesborn Wao, Helen Mahony, Ambuj Kumar, Benjamin Djulbegovic

**Affiliations:** 1 Department of Internal Medicine, Division of Evidence Based Medicine and Outcomes Research, Morsani College of Medicine, University of South Florida, Tampa, FL, USA

## Abstract

**Background:**

Systematic review (SR) of randomized controlled trials (RCT) is the gold standard for informing treatment choice. Decision analyses (DA) also play an important role in informing health care decisions. It is unknown how often the results of DA and matching SR of RCTs are in concordance. We assessed whether the results of DA are in concordance with SR of RCTs matched on patient population, intervention, control, and outcomes.

**Methods:**

We searched PubMed up to 2008 for DAs comparing at least two interventions followed by matching SRs of RCTs. Data were extracted on patient population, intervention, control, and outcomes from DAs and matching SRs of RCTs. Data extraction from DAs was done by one reviewer and from SR of RCTs by two independent reviewers.

**Results:**

We identified 28 DAs representing 37 comparisons for which we found matching SR of RCTs. Results of the DAs and SRs of RCTs were in concordance in 73% (27/37) of cases. The sensitivity analyses conducted in either DA or SR of RCTs did not impact the concordance. Use of single (4/37) versus multiple data source (33/37) in design of DA model was statistically significantly associated with concordance between DA and SR of RCTs.

**Conclusions:**

Our findings illustrate the high concordance of current DA models compared with SR of RCTs. It is shown previously that there is 50% concordance between DA and matching single RCT. Our study showing the concordance of 73% between DA and matching SR of RCTs underlines the importance of totality of evidence (i.e. SR of RCTs) in the design of DA models and in general medical decision-making.

## Background

Medical decision-making requires a comprehensive analysis of benefits and harms associated with available treatment options. Randomized controlled trials (RCTs), and in turn systematic reviews (SRs) of RCTs, are considered the reference standard in resolving treatment uncertainties [1,2]. However, in many instances RCTs and in turn SR of RCTs lack the power and long duration of follow up needed to assess the long-term outcome estimates [3]. Decision analysis (DA) can be used to provide the required estimates to inform medical decision-making [4].

Decision analysis models have been used in medical-decision making since 1972 [5-7]. For a DA model to be useful and applicable it should reflect real problems of patients and data on clinical outcome probabilities should be generated using a systematic approach. While DAs can allow users to make informed decisions when confronted with difficult clinical scenarios, their oversimplification of real world scenarios can be problematic [8]. DA models are often based on data derived from empirical studies with short-term follow up and the biases from these studies influence modeled outcomes [3]. Although there are guidelines for assessing the usefulness of DA and their role in medical decision-making [9,10] very few studies have assessed the soundness of DAs. That is, how often DA results agree with findings of matching RCTs or SR of RCTs (published after the DA) has not been comprehensively investigated. We are aware of two studies that have assessed the soundness of DA using subsequent clinical study results [11,12]. The study by Bress et al. conducted in the field of infectious diseases determined that findings of DAs were in concordance with findings of clinical studies (including RCTs and observational studies) in 75% of the cases assessed [11]. We have previously shown that findings of DAs and matching RCTs are in concordance only 50% of the time [12]. However, it is not known how often findings of DAs correspond with matching SRs of RCTs. Accordingly, the objective of this study is to assess how often findings of DAs are in concordance with matching SRs of RCTs.

## Methods

### Eligibility criteria

Decision analyses comparing two or more treatments were eligible for inclusion in this study. Systematic reviews of RCTs published after the matching DA models were included. That is, SR of RCTs published before the matching DA was not included in this study. If a matching SR for an included DA model was not found, the DA was excluded for the study.

### Information sources

We searched PubMed and Cochrane library for identifying DA and matching SR of RCTs.

### Search strategy: decision analysis papers

“Decision Support Techniques” was introduced as medical subject heading term in year 2000. Hence, we searched PubMed (Medline) for DAs from 01/2000 to 12/2008 for DAs using the following search strategy: (“Decision Support Techniques”[Mesh] OR (“Decision Making”[Mesh]) OR (“Decision Analysis”) AND (“Therapeutics”[Mesh] OR “therapy ”[Subheading] OR “Treatment Outcome” [Mesh] OR “Therapies, Investigational”[Mesh])).

### Search strategy: systematic reviews of RCTs

We searched PubMed and Cochrane library for systematic reviews (SRs) of RCTs that matched the identified DAs based on patient population (P), intervention (I), control (C) and outcome (O) (PICO) criteria. Clinical Queries search strategies in Pubmed which have been updated based on the filter developed by Haynes et al. were also utilized to search for SRs of RCTs matching the DAs [13]. Systematic reviews of RCTs published after the matching DA models were included. Keywords from DA intervention and control arms were used and, if necessary, search returns were narrowed by using keywords from the DA patient population.

### Study selection

Abstracts of all the identified studies were reviewed by one reviewer (RM) for inclusion according to the pre-determined criteria. In addition, 2 reviewers (BD and AK) randomly selected and reviewed 15% of the citations for inclusion to assess for accuracy. Another set of reviewers (HG and HW) reviewed list of all citations to identify matching SRs of RCTs for the included DAs. The list of matched SR of RCTs and DA were further confirmed independently by 2 reviewers (RM and AK). Any disagreements in the selection process of DA and matching SR of RCTs were resolved by consensus.

### Data extraction

Data were extracted from each included DA and SR of RCTs using a standardized data extraction form. Data were extracted on PICO elements from all DAs and matching SRs of RCTs. From each DA we also extracted data on whether single versus multiple data sources were used the design of DA model. From each included SR of RCTs data were also extracted on the number of RCTs included, sample size and year of publication. Data abstraction from included DAs was done by one reviewer (RM) and from SR of RCTs by two reviewers (HG and HW). Senior reviewers (BD and AK) randomly selected and reviewed 15% of the extracted data from included studies to assess for accuracy.

### Outcomes

#### Matching of DA and SR of RCTs

Abstracts of identified SRs of RCTs were reviewed by two reviewers (HW and HG) independently to determine the degree of matching based on PICO elements as follows: Overall the matching of DA and SR was done for all individual PICO elements at 3 levels classified either as optimum, broad or broadest match. First the match was done at participant/patient population level followed by intervention(s), control and outcome(s). If the initial match at participant/disease level was not achieved, the DA was excluded from the review. PICO elements of SR of RCTs were considered an *optimum* match to a DA if it involved same PICO elements. The PICO elements of SR of RCTs were considered a *broad* match to a DA if it involved similar PICO elements. The PICO elements of SR of RCTs were considered a *broadest* match to a DA if it involved only slightly similar PICO elements.

Examples of optimum, broad and broadest match are shown in Table 1. In situations where multiple matches were found, the most recently published SR of RCTs was chosen.

**Table 1:** Examples of matching based on PICO elements

**PICO element**	**Decision analysis**	**Systematic review of RCTs**	**Matching category**
Participant (P)	“premenopausal women with newly diagnosed hormone responsive breast cancer” [14]	“premenopausal women with early breast cancer which was responsive for estrogen receptor” [15]	Optimum
	“women with breast cancer” [16]	“women with early stage breast cancer” [17]	Broad
	“ high risk women seeking prophylactic mastectomy” [18]	“women with invasive breast cancer” [19]	Broadest
Intervention (I)/Control (C)	“breast conserving surgery” (intervention) and “mastectomy” (control) [16]	“breast conserving surgery” (intervention) and “mastectomy” (control) [20]	Optimum
	“medical ovarian suppression or surgical ovarian suppression” (intervention). [14]	“medical ovarian suppression” (intervention) [15]	Broad
	“conservation therapy (medical and other methods)” (control) [21]	“medical therapy” for patients with non-acute coronary artery disease (control) [22]	Broad
	“surgery” (intervention) [23]	“laser excision” in patients with glottic cancer (intervention) [20]	Broadest
Outcome (O)	“overall survival” [14]	“overall survival” [15]	Optimum
	“complications” [18]	“morbidity” [19]	Broad
	“breast cancer mortality” [24]	“all-cause mortality” [25]	Broadest

#### Concordance and impact of sensitivity analysis

It is well established that majority of the DAs conduct and report sensitivity analyses and the final outcomes of the DA model may be influenced by these sensitivity analyses. Hence, to assess the impact of the DA outcomes after these sensitivity analyses on the concordance or discordance with the findings of matching SR of RCTs we extracted data on the sensitivity analyses. Specifically, for each included DA and SR of RCTs, two review authors (HW and HG) independently extracted data on the author’s overall conclusion (indicating which treatment was better); whether author’s conclusion changed after sensitivity analysis and whether the conclusion(s) of DA and SR of RCTs agreed or disagreed. Discrepancies between the two reviewers’ judgments were resolved by discussion and mutual consensus with other reviewer (RM).

### Statistical analysis

We used descriptive statistics to report concordance or discordance between results of DA and SR of RCTs. Impact of variables that are used in the construction of DA model on concordance between DA and matching SR of RCTs was assessed by Fishers’ exact test. The impact of sample size of SR of RCTs on concordance of findings between DA and SR of RCTs was tested using Kruskal–Wallis one-way analysis of variance test [26].

## Results

### Study selection

**Figure 1:**
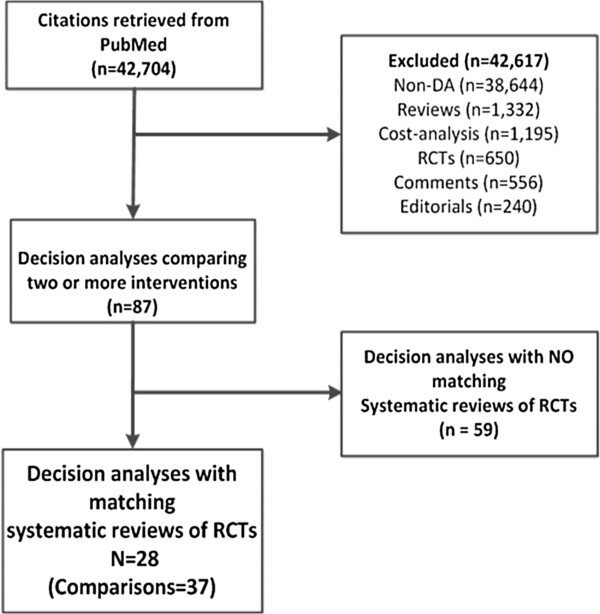
Flow diagram depicting study selection process.

The PubMed search for DAs yielded 42,704 citations (Figure 1). We excluded 42,617 studies after reviewing abstracts and found 87 studies that used DA modeling to compare two or more interventions. We found matching SR of RCTs for 32% (28/87) of DAs. These 28 DAs included 37 comparisons for which we found a matching SR of RCTs.

### Characteristics of included DAs and SRs

Infection (11/37) and cancer (10/37) were the most frequently studied diseases using DA modeling. The included DAs investigated effects of medications in 56% (21/37) of cases compared with surgical interventions in 38% (14/37) of cases (Table 2). Ninety five percent (35/37) of DAs did not collect any primary data and used data published in the literature in designing DA model. Only 5% (2/37) of DA models used a systematic approach (e.g. meta–analysis) to data collection. Similarly, 5% (2/37) of DA models used expert opinion in designing the DA model. Ninety seven percent (36/37) of DAs conducted sensitivity analysis while 51% (19/37) of included SRs of RCTs conducted sensitivity or sub-group analysis. The median sample size of included SR of RCTs was 2610 (range: 42 to 32523).

**Table 2:** Characteristics of decision analysis and systematic review of RCTs

**Characteristic**	**N (%)**
**DA Disease category**	
Infectious diseases	11 (29.7)
Cancer	10 (27)
Cardiovascular disease	5 (13.5)
Venous ailments	3 (8.1)
OB/GYN	2 (5.4)
Other	6 (16.2)
Pressure ulcers	2 (2)
Crohns disease	1 (1)
Obesity	1 (1)
Achilles Tendon Rupture	1 (1)
Anti-phospholipid antibody syndrome	1 (1)
**DA intervention type**	
Medication	21 (56)
Surgery	14 (38)
Abstaining from breast feeding	1 (3)
Early weaning from breast feeding	1 (3)
**DA control type**	
Medication	18 (48.6)
Observation	10 (27)
Surgery	5 (13.5)
Breast feeding	2 (5.4)
Radiation	1 (2.7)
Placebo	1 (2.7)
**SR of RCTs characteristic**	
Median sample size (range)	2610 (42–32523)

### Matching between DA and SR of RCTs

As summarized in Table 3 the match between DA participant characteristics with SR of RCTs was considered optimum in 57% (21/37), broad in 21% (8/37) and broadest in 18% (8/37) of cases, respectively. The match for interventions studied in DAs with interventions in the SR of RCTs was optimum in 95% (35/37) and broad and broadest in 1/37 cases each. Similarly, the matching of controls in DA with the controls used in the SR of RCTs was optimum in 92% (34/37), broad in 5% (2/37) and broadest in 3% (1/37) of cases (Table 3).

**Table 3:** Degree of matching between DA and SR of RCTs

**Category**	**N (%)**
**Patient**	
Matching with optimum criteria	21 (56.8)
Matching with broad criteria	8 (21.6)
Matching with broadest criteria	8 (21.6)
**Intervention**	
Matching with optimum criteria	35 (94.6)
Matching with broad criteria	1 (2.7)
Matching with broadest criteria	1 (2.7)
**Control**	
Matching with optimum criteria	34 (91.9)
Matching with broad criteria	2 (5.4)
Matching with broadest criteria	1 (2.7)

### Concordance between findings of DA and SR of RCTs

**Figure 2:**
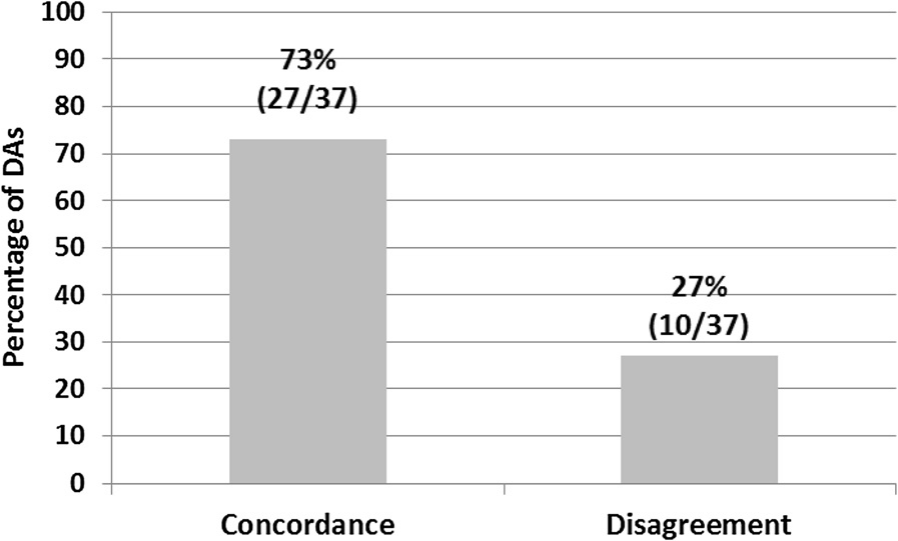
Concordance between findings of decision analysis (DAs) and systematic review of RCTs.

Overall, the findings of the DAs and the SRs of RCTs were in concordance in 73% (27/37) of cases. Twenty-seven percent (10/37) of the SR of RCTs findings were discordant with the findings of the DA (Figure 2).

Out of the 21 pairs of DA and SR with the optimum match of the patient characteristics 66% (14/21) of the DA findings were in concordance with the findings of the matching SR of RCTs. Out of the 8 pairs of DA and SR with the broad match of the patient characteristics 87% (7/8) of the DA findings were in concordance with the findings of the matching SR of RCTs. Out of the 8 pairs of DA and SR with the broadest match of the patient characteristics 75% (6/8) of the DA findings were in concordance with the findings of the matching SR of RCTs. There was no association between degree of matching based on patient characteristics and concordance of findings of DAs and SR of RCTs (p = 0.52). Similarly, there was no association between degree of matching based on intervention characteristics (p = 0.21) and control characteristics (p = 0.17) and concordance of findings of DAs and SR of RCTs.

The majority of the sensitivity analyses conducted in DA (33/37) did not impact the concordance between findings of DA and matching SR of RCTs. In three cases, the findings of DA and matching SR of RCTs were similar after the sensitivity analysis [14,16,27].

### Impact of decision analysis design attributes on concordance

The findings of DAs using multiple data sources were more likely to be concordant with matching SRs of RCTs than DAs using single data source and this association reached statistical significance (p = 0.05) (Table 4). Incorporation of data from meta-analysis (p = 1.00), use of expert opinion (p = 0.06) and primary versus secondary data collection (p = 0.47) in the design of DA model did not have any impact on concordance between findings of DA and SR of RCTs (Table 4). It is important to note that, the meta analysis used to inform the design of the DAs were obviously published before the DA model and were not used for matching the results of DAs and SRs. The distribution of sample size of SR of RCTs was similar across the SRs which were in concordance with the findings of their matched DAs compared with SRs with the findings discordant with their matched DAs (p = 0.78).

**Table 4:** Impact of DA design attributes on concordance between findings of DA and SR of RCTs

**Category**	**Concordance N (%)**	**Disagreement N (%)**	**P-value**
**Data source**			
Single data source	1(25)	3(75)	0.05
Multiple data source	26(79)	7(21)	
**Data from meta-analysis used**			
Yes	2(100)	0(0)	1.0
No	25(71)	10(29)	
**Expert opinion used**			
Yes	0(0)	2(67)	0.06
No	27(77)	8(23)	
**Primary data collection undertaken**			
Yes	1(50)	1(50)	0.47
No	26(74)	9(26)	

## Discussion

### Summary of evidence

To our knowledge, this is the first SR to date comparing the results of DAs with matching SRs of RCTs. The findings show that there is high level of concordance between findings of DA and matching SRs of RCTs and use of multiple sources of data in decision analyses appears to increase the predictive value of DA.

Comparative effectiveness research (CER) is gaining popularity and employs assessment of multiple interventions by comparing their long-term outcomes. However, in many instances the RCTs and other studies that are used in CER lack the power and long duration of follow up needed to assess the long-term outcome estimates [3]. Over past 39 years, DA has been applied to a variety of clinical problems to provide these much desired estimates to improve clinical decision making. However, the concordance between findings of empirical efficacy studies that are used for decision making (i.e. RCTs) and DA findings is not known. This largest SR to date shows concordant findings of DAs and matching SRs of RCTs in 73% of cases. The findings from our study also emphasizes on the importance of SR as we have previously shown that results of DAs and matching single RCT disagree about only 50% of the time [12].

Our findings are also in line with the other research study on the topic. The study by Bress et al. focused on infectious diseases and assessed the concordance of DA findings compared with subsequent clinical study results [11]. The study by Bress et al. determined that findings of DAs were in concordance with findings of clinical studies in 75% of the cases assessed [11]. However, the study by Bress et al. was limited to the field of infectious diseases and compared findings of DAs with either RCTs or observational studies [11]. Moreover, this study by Bress et al. did not comprehensively report the impact of DA design attributes and sample size of matching SRs on concordance between findings of DAs and SRs. We also explored the reasons for concordance and discordance between findings of DAs and matching SRs of RCTs employing multiple analyses. Specifically, we investigated the impact of DA design factors and sample size of matching SR of RCTs on concordance and discordance between findings of DA and SR of RCTs. Our results indicate that none of the attributes except use of single versus multiple data source in the design of DA models is significantly associated with concordance of findings between DA and matching SR of RCTs. Sample size of matching SR of RCTs did not have any impact on concordance and discordance between findings of DAs and SR of RCTs either. Another factor that may impact concordance between findings of DA and SR is the degree of matching between DA and SR PICO attributes which was performed in our study. In our study, the intervention and controls studied in DAs closely matched with SRs in majority of the cases. However, patient population enrolled in DAs closely matched with SR in 57% of cases. Nonetheless, the degree of patient population matching did not have any impact on concordance between findings of DA and SR of RCTs.

### Limitations

Our study has some limitations. There were a relatively small number of published DAs and an even smaller number with matching SR of RCTs. However, since DAs are mostly conducted when a RCT is not available this was expected. Nonetheless, our findings are based on small sample size (n = 37) and hence should be interpreted with caution. We did not search for unpublished DAs or SR of RCTs. As noted by Bress et al., our literature search also could not distinguish DA from other study designs. If “decision analysis” were a MeSH term, such searches would be more efficient and reproducible [11]. As a result, we reviewed large volume of citations (n = 42,704) and hence our search is not updated since 2008.

## Conclusions

Our results show the high concordance of findings of current DA models compared with findings of SR of RCTs. Moreover, our results outline the importance of SR of RCTs compared with a single RCT in medical decision making. That is, the concordance between DA findings and matching single RCT findings was only 50% [12] but the concordance between findings of DA and matching totality of evidence (i.e. SR of RCTs) was 73%. This underscores the importance of use of research synthesis in medical decision making.

Our study findings are important and informative to the design of DA models. It is known that, unless all clinically important factors have been included, the DA lacks sufficient representativeness to be clinically useful [7,28-30]. Moreover, DA designs need to follow a consistent set of best practices for selecting (estimates from SR/MA rather than individual studies), adjusting for bias and incorporating empirical evidence [3,31-33]. Our findings further highlight the need of further investigation of the impact of DA design attributes such as use of meta-analysis data and data from multiple sources on clinical rationality of DA models. Investigation of influence of DA design attributes on usefulness of DA model in decision making will further improve use of DAs in healthcare decision making and policy development.

## Competing interests

All authors had full access to all the data in the study and take responsibility for the integrity of the data and the accuracy of the data analysis. All authors declare no conflict of interest.

## Authors’ contributions

RM conducted the search, identified all the DA models, and extracted the data from DA models. HW and HM extracted data from SR of RCTs. AK and BD randomly checked the accuracy of extracted data. RM wrote the first draft of the manuscript. HW, HM, AK and BD contributed to the final draft of this manuscript. All authors read and approved the final manuscript.

## Pre-publication history

The pre-publication history for this paper can be accessed here:

http://www.biomedcentral.com/1472-6947/14/57/prepub
